# Episodic Specificity in Acquiring Thematic Knowledge of Novel Words from Descriptive Episodes

**DOI:** 10.3389/fpsyg.2017.00488

**Published:** 2017-04-06

**Authors:** Meichao Zhang, Shuang Chen, Lin Wang, Xiaohong Yang, Yufang Yang

**Affiliations:** ^1^CAS Key Laboratory of Behavioral Science, Institute of PsychologyBeijing, China; ^2^University of Chinese Academy of SciencesBeijing, China; ^3^Department of Psychology, Zhejiang Normal UniversityJinhua, China

**Keywords:** word learning, ERP, N400, LPC, thematic relation, episode

## Abstract

The current study examined whether thematic relations of the novel words could be acquired via descriptive episodes, and if yes, whether it could be generalized to thematically related words in a different scenario. In Experiment 1, a lexical decision task was used where the novel words served as primes for target words in four conditions: (1) corresponding concepts of the novel words, (2) thematically related words in the same episodes as that in learning condition, (3) thematically related words in different episodes, or (4) unrelated words served as targets. Event related potentials elicited by the targets revealed that compared to the unrelated words, the corresponding concepts and thematically related words in the same episodes elicited smaller N400s with a frontal-central distribution, whereas the thematically related words in different episodes elicited an enhanced late positive component. Experiment 2 further showed a priming effect of the corresponding concepts on the thematically related words in the same episodes as well as in a different episode, indicating that the absence of a priming effect of the learned novel words on the thematically related words in different episode could not be attributed to inappropriate selection of thematically related words in the two conditions. These results indicate that only the corresponding concepts and the thematically related words in the learning episodes were successfully primed, whereas the thematic association between the novel words and the thematically related words in different scenarios could only be recognized in a late processing stage. Our findings suggest that thematic knowledge of novel words is organized via separate scenarios, which are represented in a clustered manner in the semantic network.

## Introduction

One way of conceptual organization is through thematic relations, that is their participation in the same scenario or event (e.g., *car* and *garage*, Mirman and Graziano, 2011, 2012), instead of perceptual features. Thematic relations highlight the co-occurrence or interaction between concepts in “temporal, spatial, causal, or functional relations” (Lin and Murphy, 2001). Typically, concepts sharing a thematic relation perform complementary roles in the same event (Estes et al., 2011). Therefore, thematic relation refers to the complementary roles of concepts in the same events. Thematic relations have been found to be an important way to organize conceptual knowledge (Sachs et al., 2008a,b; Estes et al., 2011; Schwartz et al., 2011), and recognized as a critical part of language comprehension (Gagné and Shoben, 1997; Hare et al., 2009; Matsuki et al., 2011; Isberner and Richter, 2013).

Thematic relations are essential for making sense of individual experience (Collins and Loftus, 1975; Drews, 1987; Lin and Murphy, 2001) and predicting future similar events (Markman, 1981). For example, a cake and candles typically appear at a birthday party, that is, there is a thematic relation among cake, candles, and birthday. When we see a cake and candles in a new scenario, we may readily infer that a birthday party is taking place. Instead, the features of the cake and candles are irrelevant for you to figure out what is happening around you. Therefore, thematic relations play an important role in representing and organizing our knowledge of events and situations (Murphy, 2010).

Thus far, very little is known about how thematic relations and episodes are used to establish thematic relationships for novel words. Some researchers have suggested that learners could efficiently utilize the thematic relations in learning (Tinkham, 1997). They investigated the influence of thematic clustering on the acquisition of new vocabulary. The thematically clustered words were associated with a shared thematic concept in one theme (e.g., “beach,” “sunny,” and “swim” are in the theme of someone going to the beach and swimming in a sunny day), whereas three unassociated words served as the baseline (e.g., fork, court, brave). Participants were asked to learn novel words that were paired with the words listed above. They found that the acquired novel words which were thematically related could be better recalled than the unassociated novel words. This result suggests that the thematic clustering facilitated the acquisition of new words. However, other researchers found that bilingual adolescents had difficulty in accessing the thematic relations between words in their second language, and claimed that the thematic relations are hard to obtain in reading (Li et al., 2011). The present study further examined whether learners could easily acquire thematic knowledge of newly learned words from language context.

Given that thematically related concepts are connected via scenarios and events in the real world, it is conceivable that the thematic relations are closely linked to one’s knowledge and personal experience with objects and events (McRae et al., 1998; Lin and Murphy, 2001). In this sense, a concept can play different roles in various situations, and therefore associates to other concepts via different thematic relations. For example, “*car*” can be related to “*driver*” via the event of “driving a car,” to “*garage*” via the event of “*parking a car*,” and also to “*brush*” via the event of “*washing a car*.” Previous studies have shown that the frequency of thematic relations influenced the relational interpretation of novel compounds. For example, “*chocolate*” often represents a “*made of*” relation when it serves as a modifier in compounds (e.g., *chocolate bar*), so novel compounds using this frequent relation (e.g., *chocolate coin*) were interpreted more easily than those using infrequent relations (e.g., *chocolate magazine*) (Gagné and Shoben, 1997; Gagné and Spalding, 2004; Krott et al., 2009). In short, the thematic knowledge is shaped by individual experience with concepts in specific events. Therefore, the second research question we examined was how learning experience shapes the acquisition of thematic relations of novel words. Specifically, whether a novel word learned in one scenario could be automatically connected to another concept in a different scenario?

It has been well-established that readers can actively derive meaning of unknown words from sentence or discourse contexts (Nagy et al., 1985; Chaffin et al., 2001; Batterink and Neville, 2011), and further associate the learned novel words with other semantically related words (Borovsky et al., 2012; Chen et al., 2014). Given that thematic relations are generated in specific episodes, the learning condition in present study was to infer the meaning of a novel word from a descriptive episode. Then a semantic priming paradigm (Rossell et al., 2001) was used to examine the semantic relations between learned novel words and other known words. Participants read a prime word followed by either a real-word or a pseudoword as a target. They were asked to decide whether the second word was a real word or not as quickly as possible. Faster response to target words would indicate that the novel word could facilitate the processing of target word. In the ERP literature, effects of semantic relatedness have also been found on the N400 which is a sensitive measure of semantic relatedness. The N400 amplitude is reduced when a word was predictable or semantically related to the previous stimulus (Kutas and Hillyard, 1980; Kutas and Van Petten, 1994; Kutas and Federmeier, 2000). Therefore, the effects of semantic priming for related target words could be measured by speeded reaction times and reduced N400 amplitude relative to the unrelated targets.

Previous studies have reported robust semantic priming effects for semantically related and thematically related words (Estes and Jones, 2009; Sass et al., 2009; Jones and Golonka, 2012; Chen et al., 2013). In the present study, the newly learned words in the preceding episodes served as the prime words. The target words were: corresponding concepts (CC targets), thematically related words in the same episodes (THS targets), thematically related words in different episode (THD targets), and unrelated words (NR targets; see **Table 1** for examples). For example, when the learned novel word “*Juhui*” (corresponding to the concept “*candle*”) related to lighting in the descriptive learning episode, the THS target for “*Juhui*” (“*candle*”) was “*match*,” and the THD target was “*cake*.” A priming effect was expected for the corresponding concepts (i.e., a reduced N400 compared to the unrelated words) if participants could successfully infer the meaning of the novel words. Although the THS and THD targets were thematically related to the corresponding concepts, they belonged to different episodes. In the current study, the novel words were embedded in the learning episode that the participants utilized to infer the meaning of the novel words. The THS targets were thematically related to the learning episodes whereas the THD targets were not directly related to the learning episodes. It has been shown that the thematic knowledge is shaped by the experience with concepts in corresponding events (McRae et al., 1998; Lin and Murphy, 2001). Therefore, it was expected that the acquired thematic knowledge of the novel words was dependent on the learning experience. More specifically, participants could only obtain the thematic knowledge relevant to the learning condition. Accordingly, the priming effect would only be observed for the thematically related words in the same episodes. In order to further confirm that the different priming effects obtained in THS and THD conditions were exclusively caused by the manipulation of learning conditions, we conducted Experiment 2 to make sure that the words used in both THS and THD conditions could produce similar priming effects by the thematically related words. Therefore, Experiment 2 tested the priming effects of the corresponding concepts of the novel words (CC targets) on the THS, THD, and NR targets.

**Table 1:** Examples of the learning discourse and testing targets.

Learning discourse in the learning phase			
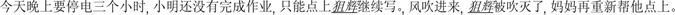			
(Tonight the electricity will be cut off for 3 h, Xiaoming hasn’t finished his homework, so he has to light up a *Juhui* to continue with the writing. The wind blew in, and the *Juhui* was blown out. His mom helped him to rekindle it.)			
**Primes and Targets in the testing phase**			

**Target conditions**	**Primes**		**Targets**

Corresponding concept (CC):	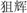 (*Ju hui*)	–	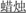 (*candle*)
Thematic-related word in the same episodes (THS):	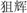 (*Ju hui*)	–	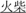 (*match*)
Thematic-related word in a different episode (THD):	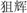 (*Ju hui*)	–	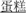 (*cake*)
Unrelated word (NR):	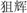 (*Ju hui*)	–	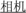 (*camera*)
Pseudoword:	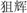 (*Ju hui*)	–	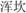 (*hun kan*)
Pseudoword:	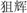 (*Ju hui*)	–	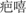 (*ba xi*)
Pseudoword:	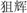 (*Ju hui*)	–	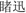 (*bi xiang*)
Pseudoword:	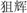 (*Ju hui*)	–	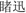 (*du xun*)


*The examples were originally in Chinese, with the critical words underlined. The English translations of the learning discourse are given below the original Chinese materials in the parenthesis. The English translations of the target words are given after the original Chinese words in the parenthesis.*

## Experiment 1

### Method

#### Participants

We recruited 32 university students (mean age 21 years, 15 males) to participate in Experiment 1. They were all right-handed native speakers of Chinese, with normal or corrected to normal vision. None of them had dyslexia or any neurological impairment. Before the experiment, they signed a written consent form. Then they were informed of the recall task after the learning phase and they were told that their compensation would be paid based on their performance. After the experiment, they were paid according to their response accuracy and recall rate in the experiment. Two participants (one male) were excluded because of frequent slow-wave drifts. Another (female) participant was excluded due to her extremely poor performance (she only recalled the meaning of one novel word out of 34 novel words in a recall task, see the *Procedure* section). Therefore, the data of 29 participants (mean age 21 years, 14 males) entered the result analysis.

#### Materials

Thirty-four two-character pseudowords (e.g., “*Juhui” for “
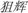
*”) served as the novel words learned in discourses. Each discourse consisted of two sentences describing an episode containing one event. The novel word was presented only once in each sentence (see **Table 1**). In order to make sure that participants could successfully infer the meaning of the novel words via the learning discourse passages, we asked 10 paid volunteers (4 males) to read these discourse passages and guess the meaning of the novel words. As expected, the participants could successfully infer the meaning of the novel words (correct rate: mean ± *SD* = 95% ± 8.3%).

The novel words were used as the prime words in the lexical decision task, followed by real-word targets or unlearned-pseudoword targets (see **Table 1**). The four conditions of the target words were: (1) the corresponding concept of each novel word (CC, e.g., *candle*), (2) the thematically related word in the same episodes (THS, e.g., *match*), (3) the thematically related word in a different episode (THD, e.g., *cake*), and (4) the unrelated word (NR, e.g., *camera*). Altogether, there were 136 novel word-target pairs and 136 novel word-pseudoword pairs. We also asked the 10 participants who rated the discourse passages to judge to what extent the THS and THD target words were related to the learning episode in a 5-point Likert scale (5 indicates the most closely related and 1 indicates unrelated). The participants rated the THS targets (mean ± *SD* = 4.19 ± 0.39) to be more related to the learning episodes than the THD targets (mean ± *SD* = 2.21 ± 0.43, *F*_(1,33)_ = 360.71, *MSE* = 0.18, *p* < 0.001, η*^2^* = 0.92). In addition, latent semantic analysis was conducted to control the semantic association between the critical words and all words within the discourse passages (website^1^, statistic cosine Similarity of LSA). The results showed that the THS and THD targets had equally low LSA semantic relatedness to words within the learning discourse passages, *F*_(1,33)_ = 0.28, *MSE* = 0.01, *p* = 0.60, η*^2^* = 0.01.

In addition, we recruited another 10 participants (5 males) to rate the thematic relatedness (i.e., to what extent the two concepts can be related by an event or a scenario) between the corresponding concepts of the novel words (i.e., the CC words) and the THS, THD, and NR targets in a 5-point Likert scale (5 indicates the most closely related, and 1 indicates unrelated). An one factor (Target condition: THS, THD, and NR) repeated ANOVA confirmed that the THS and THD targets were judged as being more thematically related to the CC words than the NR targets, and the THS and THD were equally related to the CC words, while the NR targets were not related to the CC words [*F*_(2,66)_ = 526.26, *MSE* = 0.18, *p* < 0.001, η*^2^* = 0.94; THS vs. NR: *t*_(33)_ = 35.61, *p* < 0.001; THD vs. NR: *t*_(33)_ = 29.76, *p* < 0.001; THS vs. THD: *t*_(33)_ = 1.53, *p* = 0.40].

The target words in the four conditions were matched for word frequency based on the corpus developed by Cai and Brysbaert (2010) [*F*_(3,99)_ = 0.09, *MSE* = 79.15, *p* = 0.96, η*^2^* = 0.003] as well as number of strokes [*F*_(3,99)_ = 1.55, *MSE* = 22.68, *p* = 0.21, η*^2^* = 0.05]. **Table 2** reports the linguistic properties of the stimuli. In addition, the strokes of each word and phrase prior to the novel word target in the learning discourse passages was also matched. The results revealed that there was no significant main effect of presentation position [Means (SD) of First presentation vs. Second Presentation were 22.48 (12.93) vs. 24.76 (11.92), *F*_(1,33)_ = 0.95, *MSE* = 168.27, *p* = 0.34, η*^2^* = 0.03].

**Table 2:** Means (SDs) of the stimuli properties.

Condition	Latent semantic analysis	Learning episodic relatedness	Thematic relatedness	Frequency	Number of stroke
CC:	–	–	–	7.06 (8.86)	17.71 (4.88)
THS:	0.14 (0.08)	4.19 (0.39)	4.19 (0.46)	6.21 (9.33)	15.41 (4.29)
THD:	0.13 (0.11)	2.21 (0.43)	3.99 (0.48)	6.24 (14.10)	17.32 (4.40)
NR:	–	–	1.19 (0.14)	7.00 (8.76)	16.50 (5.35)


#### Procedure

Participants were seated in a comfortable chair in front of the screen of a computer. The distance between the participants and the screen was about 80 cm. The Chinese characters (Song typeface) were presented in white color with a black background. The font size was 32 in both the learning discourse passages and the word pairs in the lexical decision task.

Similar to the learning procedure of previous ERP studies (e.g., Chen et al., 2014; Ding et al., 2017), in the current learning trial, a fixation was presented in the center of the screen lasting randomly from 800 to 1200 ms. Then the learning discourse passage was presented one phrase at a time, each one lasting for 500 ms. Two consequent phrases were separated by 300 ms blanks. The novel words (e.g., 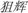) in the learning discourse passages always appeared in isolation for 1000 ms. After the last phrase, the whole learning discourse passage was presented in the screen. The participants were asked to press the space button if they had understood the discourse and learned the meaning of the novel word. A resting screen of 2000 ms was presented before the next trial began. After learning 8 or 9 novel words, the participants were allowed to have a break. Then the testing trials began. A fixation cross was presented in the center of the screen lasting randomly from 800 to 1200 ms. Then a prime word was presented for 300 ms followed by an interval of 200 ms. After that, a target word appeared for 300 ms. Once the target word appeared, the participants were asked to judge whether it was a real word or not as quickly as possible by pressing the “F” or “J” buttons on the keyboard. Then a resting screen was presented for 2000 ms before the next trial started.

We divided the 34 discourse passages and the corresponding 272 word pairs into four blocks. In each block, participants learned 8 or 9 novel words and judged 64 or 72 word pairs. The learning discourse passages were presented randomly while the word pairs were presented in a pseudo-random order. In order to ensure that the meanings of the novel words were not learned by the pairing of the corresponding concepts with the novel words in the testing phase, the novel word-CC target pairs were always presented after the THS, THD, and NR targets. Word pairs containing the same novel word were separated by at least seven other word pairs. We also made sure that the same response was not repeated for more than three times. There was a short break between blocks. In order to further test the memory performance of the participants, we asked the participants to perform a recall task after all blocks were completed. We showed the novel words to the participants and asked them to write down the corresponding concepts of the novel words.

#### Electroencephalogram (EEG) Recording and Preprocessing

The EEG was recorded from 64 Ag/AgCl electrodes in an elastic cap according to the International 10–20 system. These electrodes were referenced to the right mastoid. An electrode placed between FPz and Fz served as the ground. A supra- to suborbital bipolar montage was used to monitor the vertical eye movements and blinks. The horizontal eye movements were monitored via a right to left canthal bipolar montage. EEG data were amplified with an AC amplifier and recorded at a sampling rate of 500Hz, with a band pass filter of 0.05–100 Hz. Impedances of most electrodes were kept below 5 KΩ.

We preprocessed the raw EEG data using NeuroScan software 4.3. The ocular artifacts were automatically corrected (Semlitsch et al., 1986). The data were firstly band-pass filtered offline between 0.1 and 30 Hz and then segmented into epochs from -200 to 1000 ms relative to the novel words in the learning phase and the target words in the lexical decision task. A baseline correction was applied from 200 to 0 ms preceding the novel words and target words onset. An artifact criterion of ±80 μV was used at all scalp sites except the EOG electrodes to reject trials with excessive noise transients, after which the EEG data were re-referenced to the average of both mastoids. The average proportion of rejected trials was 5.9 and 8.2%, respectively, for the learning phase and the lexical decision task. Finally, ERPs were averaged in each condition for each participant.

#### ERP Data Analysis

**FIGURE 1:**
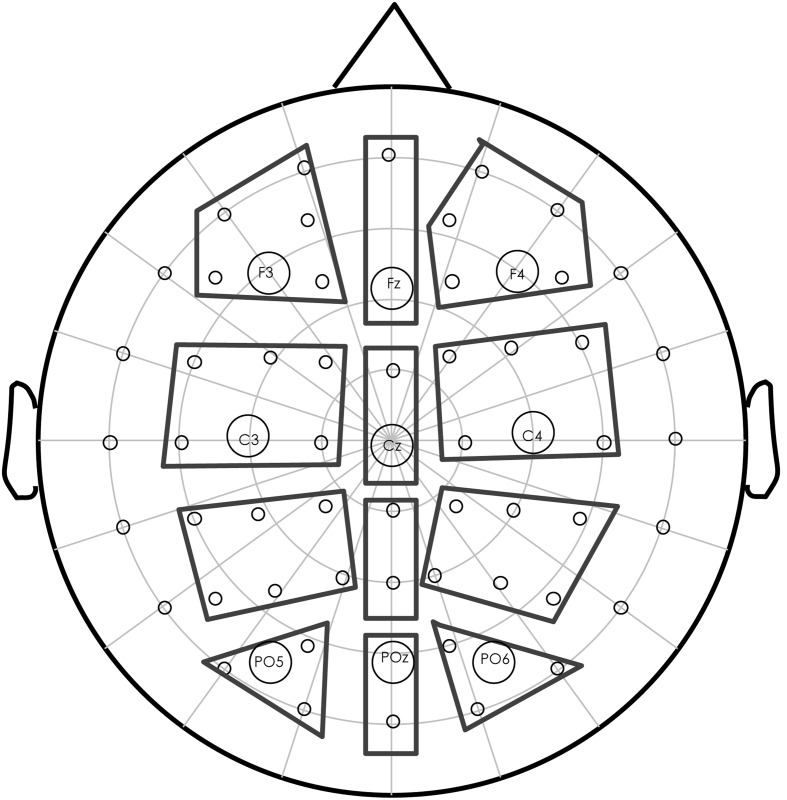
**Electrode layout on the scalp.** The electrodes selected for statistical analysis are grouped into eight regions for the lateral electrodes: left-frontal, left-central, left-parietal, left-posterior, right-frontal, right-central, right-parietal, and right-posterior. The midline electrodes were grouped into four regions: midline-frontal, midline-central, midline-parietal, and midline-posterior. The indicated electrodes (F3, Fz, F4, C3, Cz, C4, P3, Pz, and P4) were used to present the ERP waveforms.

The mean amplitude values were calculated for each participant, each condition, within each selected time window, and entered into further statistical analysis. The selected electrodes were demonstrated in **Figure 1** (enclosed in solid lines). Repeated measures ANOVAs were conducted separately for the midline and lateral electrodes. For the midline electrodes, Target condition (CC, THS, THD, and NR) and Anteriority (Frontal, Central, Parietal, and Posterior) were submitted to the ANOVAs. For the lateral electrodes, Hemisphere (Left, Right) was an additional electrodes position factor.

In the ANOVA measures, we applied the Greenhouse-Geisser correction if the degree of freedom was larger than one and the Mauchly’s sphericity test was significant. In these cases, we reported the original degrees of freedom and the corrected *p* values. In addition, when there were any interactions with the Target condition in the ANOVAs, simple effect tests and planned comparisons were conducted. The pair-wise comparisons were all adjusted by Bonferroni correction.

### Results^2^

#### Behavioral Data

##### Lexical decision task

**FIGURE 2:**
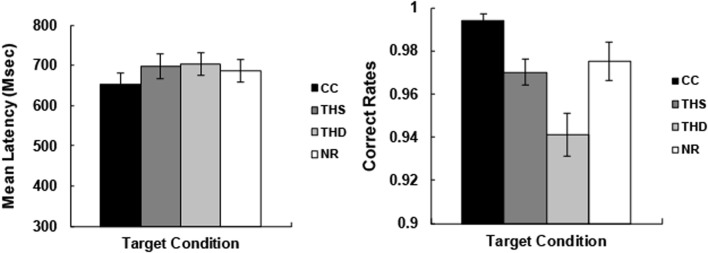
**Mean latencies of correct responses (in ms, Left panel) and correct rates (in percentage, Right panel) for target words in each condition (CC, THS, THD, and NR).** Error bars represent the standard error.

Prior to the analysis, error reactions and outlier data points (mean ± 2.5SD) were removed from the RT data (6.0% among all data). The RT results (left panel) and the accuracy results (right panel) were shown in **Figure 2**. The reaction times and accuracy were submitted to a one-way (Target condition: CC, THS, THD, NR) repeated measures ANOVA. For reaction times, we found a significant main effect of Target condition [*F*_(3,84)_ = 5.13, *p* < 0.01, η*^2^* = 0.16]. Pair-wise comparisons revealed that the responses to the CC targets were significantly faster than those of the THD targets (CC vs. THD: *t*_(28)_ = -3.22, *p <* 0.05).

The mean accuracy was 99.4, 97.0, 94.1, and 97.5%, respectively, for the CC, THS, THD, and NR targets. The repeated measures ANOVA of the four Target conditions (CC, THS, THD, NR) revealed a significant main effect [*F*_(3,84)_ = 15.46, *p* < 0.001, η*^2^* = 0.36]. Pair-wise comparisons revealed that the accuracy for the CC targets was higher than that of the THS and THD targets (CC vs. THS: *t*_(28)_ = 4.80, *p <* 0.001; CC vs. THD: *t*_(28)_ = 5.89, *p <* 0.001). In addition, the accuracy rates for the NR targets were higher than that of the THD targets (NR vs. THD: *t*_(28)_ = 3.67, *p <* 0.01).

##### Recall task

The recall accuracy for the learned novel words was 43.2% (ranging from 5.9 to 100%), suggesting that not all participants could explicitly recall the meaning of the novel words after a long delay. The large individual difference in the recall accuracy may stem from the participants’ memory strategy. The participants who exhibited excellent performance in the recall task claimed that they used associative strategy to memorize the meaning of the novel words.

#### ERP Data

##### Learning phase

**FIGURE 3:**
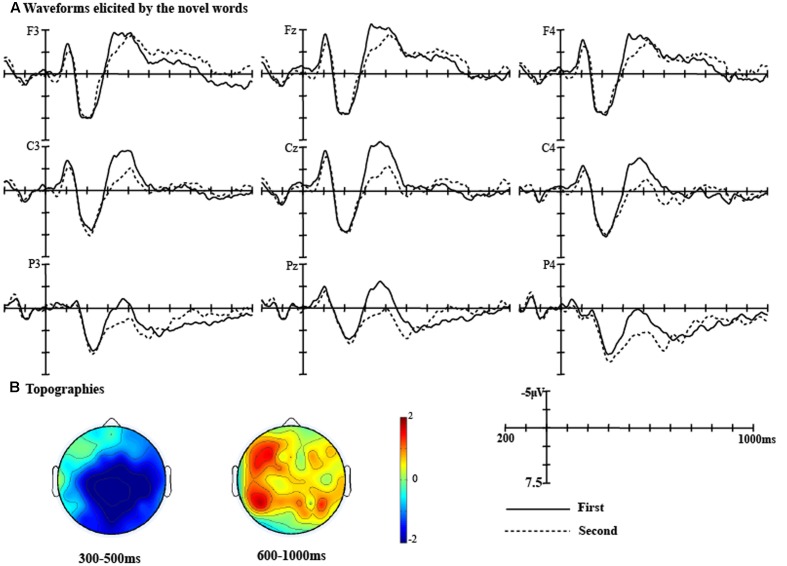
**(A)** Waveforms for the novel words in the first sentence and the second sentence were presented at F3, Fz, F4, C3, Cz, C4, P3, Pz, and P4 electrodes. **(B)** Topographies showing the average amplitude voltage differences between the novel word in the first sentence and second sentence in 300–500 ms time window and 600–1000 ms time window.

The grand average waveforms elicited by the novel words in the learning phase at different positions (First, Second) at nine representative electrodes (F3, Fz, F4, C3, Cz, C4, P3, Pz, and P4) were presented in **Figure 3**. In view of the effects, two time windows were selected for statistical analyses: (1) The standard N400 time window: 300–500 ms; (2) A late positivity time window: 600–1000 ms.

##### The N400 time window

For the lateral electrodes, the main effect of Novel word position [*F*_(1,28)_ = 16.21, *p* < 0.001, η*^2^* = 0.37] and the interaction between novel word position and Anteriority were significant [*F*_(3,84)_ = 9.08, *p* < 0.001, η*^2^* = 0.25]. The simple effect analysis revealed that the novel word presented for the second time elicited a smaller N400 than that presented for the first time over the Central (*t*_(28)_ = 3.82, *p <* 0.01), Parietal (*t*_(28)_ = 4.58, *p <* 0.001), and Posterior regions (*t*_(28)_ = 4.36, *p <* 0.001).

For the midline electrodes, the main effect of Novel word position [*F*_(1,28)_ = 15.01, *p <* 0.01, η*^2^* = 0.35] and the interaction between novel word position and Anteriority were significant [*F*_(3,84)_ = 6.39, *p <* 0.01, η*^2^* = 0.19]. The novel word presented for the second time elicited a smaller N400 than that presented in the first time over the Central (*t*_(28)_ = 4.70, *p <* 0.001) and Parietal regions (*t*_(28)_ = 5.02, *p <* 0.001).

##### The LPC time window

For the lateral electrodes, the main effect of Novel word position was significant [*F*_(1,28)_ = 20.95, *p* < 0.001, η*^2^* = 0.43]. Pair-wise comparisons revealed that the novel word presented for the first time was more positive-going than that presented for the second time.

For the midline electrodes, the novel word presented for the first time was more positive-going than that presented for the second time over all middle electrodes [*F*_(1,28)_ = 11.34, *p* < 0.01, η*^2^* = 0.29].

Overall, the novel word shown for the first time elicited a larger N400 and a larger LPC than that shown for the second time.

##### Lexical decision task

We presented the ERP responses to the target words in the lexical decision task below. Only (marginally) significant effects containing the critical manipulation (Target condition) were reported.

**FIGURE 4:**
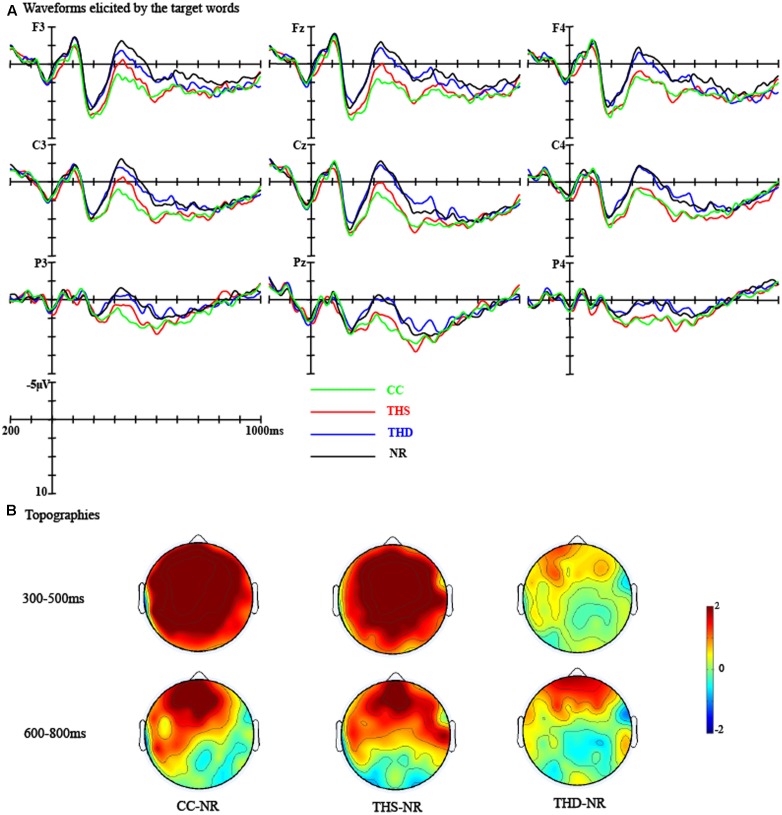
**(A)** Waveforms for CC, THS, THD, and NR targets were presented at F3, Fz, F4, C3, Cz, C4, P3, Pz, and P4 electrodes; CC, corresponding concept; THS, thematically related word in the same episodes; THD, thematically related word in a different episode; NR, unrelated word. **(B)** Topographies showing the average amplitude voltage differences between the CC, THS, THD targets, and NR targets, respectively.

The grand average waveforms elicited by the target words in different conditions at nine representative electrodes (F3, Fz, F4, C3, Cz, C4, P3, Pz, and P4) were presented in **Figure 4**. In view of the effects, four time windows were selected for statistical analyses: (1) The N1 time window: 90–130 ms; (2) The P2 time window: 180–220 ms; (3) The standard N400 time window: 300–500 ms; (4) A late positivity time window: 600–800 ms.

##### The N1 time window

For the lateral electrodes, we found a marginally significant main effect of Target condition [*F*_(3,84)_ = 2.52, *p* = 0.064, η*^2^* = 0.08]. The interaction between Target condition and Hemisphere [*F*_(3,84)_ = 3.46, *p <* 0.05, η*^2^* = 0.11] and the interaction among Target condition, Anteriority, and Hemisphere [*F*_(9,252)_ = 1.99, *p <* 0.05, η*^2^* = 0.07] were also significant. Separate ANOVAs for the left and right hemispheres revealed that the Target condition effect was only significant over Central [*F*_(3,26)_ = 3.02, *p <* 0.05, η*^2^* = 0.26] and Parietal regions [*F*_(3,26)_ = 2.95, *p* = 0.050, η*^2^* = 0.25] in the right hemisphere. Pair-wise comparisons indicated that the CC targets elicited a larger N1 than the THS targets (Central region: CC vs. THS: *t*_(28)_ = -2.92, *p <* 0.05; Posterior region: CC vs. THS: *t*_(28)_ = -3.04, *p <* 0.05).

For the midline electrodes, we found a significant main effect of Target condition [*F*_(3,84)_ = 2.72, *p* = 0.050, η*^2^* = 0.09] and a marginally significant interaction between Anteriority and Target condition [*F*_(9,252)_ = 1.90, *p* = 0.053, η*^2^* = 0.06]. Simple effect analysis revealed that the CC targets elicited a larger N1 than the THS targets over the Posterior region (CC vs. THS: *t*_(28)_ = -3.71, *p* < 0.01).

In sum, the CC targets elicited a larger N1 than the THS targets over the Central and Posterior regions in the right hemisphere, and the CC targets elicited a larger N1 than the THS targets over the Posterior region in the midline electrodes (see **Figure 4**).

##### The P2 time window

For the lateral electrodes, the interaction between Target condition and Anteriority was significant [*F*_(9,252)_ = 4.68, *p* < 0.001, η*^2^* = 0.14]. Separate ANOVAs for the Frontal, Central, Parietal and Posterior regions revealed that the Target condition effect was only significant over the Posterior region [*F*_(3,26)_ = 3.38, *p <* 0.05, η*^2^* = 0.28]. Pair-wise comparisons indicated that the CC targets elicited a smaller P2 than the THS targets (CC vs. THS: *t*_(28)_ = -2.86, *p <* 0.05). The interaction among Target condition, Hemisphere and Anteriority was also significant [*F*_(9,252)_ = 2.06, *p* < 0.05, η*^2^* = 0.07], the main effect of Target condition was significant over the Posterior region in both the left [*F*_(3,26)_ = 3.17, *p* < 0.05, η*^2^* = 0.27] and the right hemisphere [*F*_(3,26)_ = 3.49, *p* < 0.05, η*^2^* = 0.29]. Pair-wise comparisons indicated that the CC targets elicited a smaller P2 than the NR targets over the left hemisphere (CC vs. NR: *t*_(28)_ = -2.86, *p <* 0.05) and the THS targets over the right hemisphere (CC vs. THS: *t*_(28)_ = -2.80, *p* = 0.056).

For the midline electrodes, there was a significant interaction between Target condition and Anteriority [*F*_(9,252)_ = 4.69, *p* < 0.001, η*^2^* = 0.14]. Separate ANOVAs conducted to Frontal, Central, Parietal, and Posterior regions revealed that the CC targets elicited a smaller P2 than the THS targets only over the Posterior regions [*F*_(3,26)_ = 2.98, *p* = 0.050, η*^2^* = 0.26; CC vs. THS: *t*_(28)_ = -2.78, *p* = 0.064].

Overall, the CC targets elicited a smaller P2 than the NR targets over posterior regions in the left hemisphere and a smaller P2 than the THS targets over the posterior regions of the right hemisphere and midline electrodes.

##### The N400 time window

For the lateral electrodes, there was a significant main effect of Target condition [*F*_(3,84)_ = 15.81, *p* < 0.001, η*^2^* = 0.36]. The interaction between Target condition and Hemisphere was significant [*F*_(3,84)_ = 3.50, *p <* 0.05, η*^2^* = 0.11]. Separate ANOVAs for the left and right hemispheres revealed that the main effect of Target condition was more prominent over the left hemisphere [*F*_(3,26)_ = 12.10, *p <* 0.001, η*^2^* = 0.58] than over the right hemisphere [*F*_(3,26)_ = 6.91, *p <* 0.01, η*^2^* = 0.44]. Pair-wise comparisons revealed that the CC and THS targets elicited smaller N400s than the NR targets over both the left (CC vs. NR: *t*_(28)_ = 6.12, *p* < 0.001; THS vs. NR: *t*_(28)_ = 4.32, *p <* 0.01) and right hemispheres (CC vs. NR: *t*_(28)_ = 4.16, *p <* 0.01; THS vs. NR: *t*_(28)_ = 4.30, *p <* 0.01). In addition, the CC targets and THS targets elicited smaller N400s than the THD targets over both the left (CC vs. THD: *t*_(28)_ = 4.59, *p <* 0.01; THS vs. THD: *t*_(28)_ = 3.57, *p <* 0.01) and the right hemispheres (CC vs. THD: *t*_(28)_ = 3.11, *p <* 0.05; THS vs. THD: *t*_(28)_ = 3.39, *p <* 0.05). The interaction between Anteriority and Target condition was also significant [*F*_(9,252)_ = 2.83, *p <* 0.01, η*^2^* = 0.09]. Separate ANOVAs conducted to Frontal, Central, Parietal, and Posterior regions revealed that the Target condition effect was more prominent over the Frontal [*F*_(3,26)_ = 8.34, *p <* 0.001, η*^2^* = 0.49] and the Central regions [*F*_(3,26)_ = 8.60, *p <* 0.001, η*^2^* = 0.50] than over the Parietal [*F*_(3,26)_ = 7.41, *p <* 0.01, η*^2^* = 0.46] and Posterior regions [*F*_(3,26)_ = 5.54, *p <* 0.01, η*^2^* = 0.39]. Pair-wise comparisons revealed that the CC and THS targets elicited smaller N400s than the NR targets over the Frontal (CC vs. NR: *t*_(28)_ = 5.12, *p* < 0.001; THS vs. NR: *t*_(28)_ = 3.62, *p <* 0.01), Central (CC vs. NR: *t*_(28)_ = 4.79, *p <* 0.01; THS vs. NR: *t*_(28)_ = 4.67, *p <* 0.01), Parietal (CC vs. NR: *t*_(28)_ = 4.52, *p <* 0.01; THS vs. NR: *t*_(28)_ = 4.34, *p <* 0.01), and Posterior regions (CC vs. NR: *t*_(28)_ = 3.79, *p <* 0.01; THS vs. NR: *t*_(28)_ = 3.56, *p <* 0.01). In addition, the CC targets and the THS targets elicited smaller N400s than the THD targets over the Frontal (CC vs. THD: *t*_(28)_ = 3.66, *p <* 0.01; THS vs. THD: *t*_(28)_ = 2.72, *p* = 0.067), Central (CC vs. THD: *t*_(28)_ = 3.84, *p <* 0.01; THS vs. THD: *t*_(28)_ = 4.30, *p <* 0.01), Parietal (CC vs. THD: *t*_(28)_ = 3.54, *p <* 0.01; THS vs. THD: *t*_(28)_ = 3.36, *p <* 0.05), and Posterior regions (CC vs. THD: *t*_(28)_ = 3.79, *p <* 0.01; THS vs. THD: *t*_(28)_ = 3.56, *p <* 0.01).

For the midline electrodes, we found a significant main effect of Target condition [*F*_(3,84)_ = 15.55, *p* < 0.001, η*^2^* = 0.36] and an significant interaction effect between Anteriority and Target condition [*F*_(9,252)_ = 2.69, *p <* 0.01, η*^2^* = 0.09]. Separate ANOVAs conducted to Frontal, Central, Parietal, and Posterior regions revealed that the Target condition effect was more prominent over the Frontal [*F*_(3,26)_ = 9.99, *p <* 0.001, η*^2^* = 0.54] and the Central regions [*F*_(3,26)_ = 8.50, *p <* 0.001, η*^2^* = 0.50] than over the Parietal [*F*_(3,26)_ = 6.51, *p <* 0.01, η*^2^* = 0.43] and Posterior regions [*F*_(3,26)_ = 5.47, *p <* 0.01, η*^2^* = 0.39]. Pair-wise comparisons revealed that the CC and THS targets elicited smaller N400s than the NR targets over the Frontal (CC vs. NR: *t*_(28)_ = 5.48, *p* < 0.001; THS vs. NR: *t*_(28)_ = 4.04, *p <* 0.01), Central (CC vs. NR: *t*_(28)_ = 4.99, *p <* 0.001; THS vs. NR: *t*_(28)_ = 4.44, *p <* 0.01), Parietal (CC vs. NR: *t*_(28)_ = 4.20, *p <* 0.01; THS vs. NR: *t*_(28)_ = 4.12, *p <* 0.01), and Posterior regions (CC vs. NR: *t*_(28)_ = 3.51, *p <* 0.01; THS vs. NR: *t*_(28)_ = 3.64, *p <* 0.01). In addition, the CC and the THS targets elicited smaller N400s than the THD targets over the Frontal (CC vs. THD: *t*_(28)_ = 4.18, *p <* 0.01; THS vs. THD: *t*_(28)_ = 2.97, *p <* 0.05), Central (CC vs. THD: *t*_(28)_ = 3.89, *p <* 0.01; THS vs. THD: *t*_(28)_ = 3.69, *p <* 0.01), Parietal (CC vs. THD: *t*_(28)_ = 3.16, *p <* 0.05; THS vs. THD: *t*_(28)_ = 2.89, *p <* 0.05), and Posterior regions (CC vs. THD: *t*_(28)_ = 3.05, *p <* 0.05; THS vs. THD: *t*_(28)_ = 3.30, *p <* 0.05).

Overall, the CC and THS targets elicited smaller N400s than the NR targets over the whole scalp (as shown in **Figure 4**). In addition, the CC targets and THS targets elicited smaller N400s than the THD targets over the whole scalp.

##### The LPC time window

For the lateral electrodes, the interaction between Target condition and Hemisphere [*F*_(3,84)_ = 3.70, *p <* 0.05, η*^2^* = 0.12] as well as the interaction between Target condition and Anteriority [*F*_(9,252)_ = 2.78, *p <* 0.01, η*^2^* = 0.09] were significant. Separate ANOVAs conducted to left and right regions separately revealed no significant main effect of Target condition (*ps >* 0.10). Separate ANOVAs conducted to Frontal, Central, Parietal, and Posterior regions revealed that the main effect of Target condition was only significant over the Frontal region [*F*_(3,26)_ = 3.54, *p <* 0.05, η*^2^* = 0.29]. Pair-wise comparisons revealed that the CC Targets and THD targets elicited larger LPCs than the NR targets (CC vs. NR: *t*_(28)_ = 2.99, *p* < 0.05; THD vs. NR: *t*_(28)_ = 2.96, *p* < 0.05).

For the midline electrodes, the interaction between Target condition and Anteriority was also significant [*F*_(9,252)_ = 2.62, *p <* 0.01, η*^2^* = 0.09]. Separate ANOVAs conducted to Frontal, Central, Parietal, and Posterior regions revealed that the main effect of Target condition was only significant over the Frontal region [*F*_(3,26)_ = 4.91, *p <* 0.01, η*^2^* = 0.36]. Pair-wise comparisons revealed that the CC Targets and THD targets elicited larger LPCs than the NR targets (CC vs. NR: *t*_(28)_ = 3.96, *p* < 0.01; THD vs. NR: *t*_(28)_ = 2.85, *p* = 0.051).

Overall, the CC targets and THD targets elicited larger LPCs than the NR targets over the Frontal region.

### Discussion

Experiment 1 at first examined whether the thematic relations of novel words could be acquired from descriptive episodic discourse, and if yes, whether it could be generalized to thematically related words in a different scenario. During the learning phase, the novel words showed reduced N400 and LPC when they were presented for the second time. Given that the novel words were unknown words for the participants in the first encounter, they could only utilize the information from discourse to infer the meaning of the novel words. When the novel words were presented for the first time, the participants tried to keep the novel words in their working memory meanwhile retrieve episodic information from long term memory to infer the meaning of the novel words. When the novel words were shown for the second time, the information from the discourse further helped the participants to confirm the meaning of the novel words. The successful inference might have made it much easier to integrate the novel word into the episodic discourse. These results suggest that acquiring a novel concept may involve a constant construction of the meaning.

In the lexical decision task, the semantic representation of the learned novel words was tested. The novel words showed strong priming effects for the CC target words and the THS target words, as indicated by smaller N400s compared to the NR target words. It is worth noting that the N400 effect has a central-frontal topographic distribution, while the typical N400 effect has a posterior-parietal distribution (Kutas and Hillyard, 1989; Kutas and Federmeier, 2000). Our findings are consistent with a previous study by Mestres-Missé et al. (2007). They also found that the real words and novel words elicited N400 effects, respectively, over right parietal regions and frontocentral regions. The central-frontal negativity elicited by the learned novel word could be interpreted as an fN400 effect, which has been often reported in learning studies (Perfetti et al., 2005; Yum et al., 2014). The topographic difference may reflect a different neural network that was recruited when processing the real words and the newly learned words. In the novel-words condition, the associative relations between the novel words and the pre-existing words were still relatively weak, requiring more effort to be retrieved from the semantic network. In contrast, the real words and other pre-existing words have stable associations with each other in the semantic network, so they could be retrieved automatically. The involvement of cognitive effort might have activated the central-frontal cognitive network, inducing a central-frontal distributed fN400 effect. In addition, fN400 also refers to the early midfrontally distributed difference between ERPs elicited by old and new items, which operates in a way consistent with a neural marker of familiarity-based recognition (Woodruff et al., 2006; Yu and Rugg, 2010). During the inference process, although the CC and THS targets were not directly presented, they might be activated due to their high relatedness with other information presented in the same episodes. Then during the testing phase, the CC and THS targets were more familiar relative to NR targets, resulting in fN400 effects.

However, there was no significant difference between the THD targets and the NR targets in N400 amplitude, suggesting an absence of a priming effect from the novel words to the THD targets. Interestingly, the THD targets elicited a larger late positive component (LPC) comparing to the NR words over the frontal region. Since the LPC component has been related to conscious awareness of semantic relationship between primes and targets at a late processing stage (Bouaffre and Faita-Ainseba, 2007), the LPC effect observed for the THD targets might indicate that the participants were aware of the thematic associations between the novel words and the thematically related words from different scenarios even though the thematic relations could not be immediately generalized to other episodes.

Overall, the results indicate that participants successfully mapped the novel words to the corresponding concepts. The thematic relations of the novel words could only be built for the thematically related words appearing in the same episodes as the learning one, but not applicable to the words thematically related but from a different episode.

## Experiment 2

Experiment 1 revealed that the novel words could prime the CC and THS targets but not the THD targets, suggesting that the learned novel words were integrated into semantic representations by establishing thematic relations with the words in the same scenario. Thus, the thematic relations of the novel words cannot be generalized to the thematically related words from a different scenario. However, an alternative explanation is that the lack of the priming effect from the novel words to the THD targets may be solely due to the selection of different thematically related words in the THS and THD conditions. As shown in **Table 2**, the thematic relatedness between the novel words and the THD targets seems to be weaker than that between the novel words and the THS targets. For this reason, Experiment 2 was conducted to test the priming effect from the corresponding concepts of the novel words in Experiment 1 to the THS, THD, and NR targets.

### Method

#### Participants, Materials, and Procedure

Another 24 university students (mean age 22.29 ± 1.43 years, 12 males) were recruited to participate in the EEG experiment. We selected the participants according to the same criteria as Experiment 1. Three participants (three males) were excluded because of frequent slow-wave drifts. Therefore, the data of 21 participants (mean age 22.10 ± 1.37 years, 9 males) entered the result analyses. The design, materials, and procedure were identical to the lexical decision task of Experiment 1, except that the corresponding concepts of the novel words (e.g., *candle*) were used as the prime words. The thematically related words in the same episodes (THS, e.g., *match*), the thematically related words in a different episode (THD, e.g., *cake*) and unrelated word (NR, e.g., *camera*) were used as target words. The latent semantic analysis was also conducted to control the semantic association between the THS and the THD targets with corresponding concepts. The results showed that there was no significant difference between the THS [Mean (*SD*) = 0.23 (0.24)] and the THD targets [Mean (*SD*) = 0.15 (0.14), *F*_(1,26)_ = 1.99, *MSE* = 0.04, *p* = 0.17, η*^2^* = 0.07].

#### EEG Recording, Preprocessing, and Data Analysis

The EEG recording, preprocessing, and data analysis were identical to Experiment 1. The average proportion of rejected trials was 9.3% for the lexical decision task. ERPs were averaged in each condition for each participant. The mean amplitude values were calculated for each participant, each condition, within the selected time window, and entered into further statistical analysis. Repeated measures ANOVAs were conducted separately for the midline and lateral electrodes. For the midline electrodes, Target condition (THS, THD, and NR) and Anteriority (Frontal, Central, Parietal, and Posterior) were submitted to the ANOVAs. For the lateral electrodes, Hemisphere (Left, Right) was an additional electrodes position factor.

### Results

#### Behavioral Data

**FIGURE 5:**
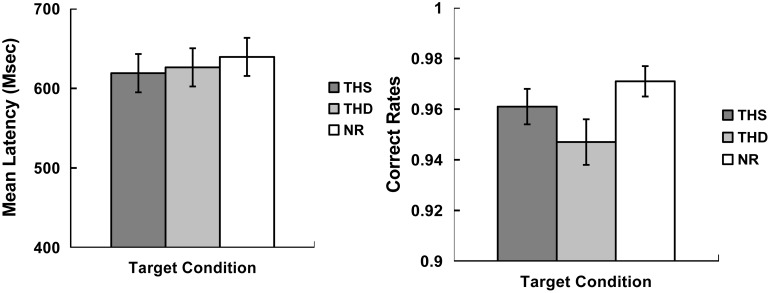
**Mean latencies of correct responses (in ms, Left panel) and correct rates (%, Right panel) for target words in each condition (THS, THD, and NR).** Error bars represent the standard error.

Similar to Experiment 1, outliers and error reactions were removed from the RT data (7.1% among all data). The RT results (left panel) and accuracy results (right panel) were shown in **Figure 5**. The reaction times and accuracy were submitted to a one-way (Target condition: THS, THD, NR) repeated measures ANOVA. For reaction times, we found a significant main effect of Target condition: *F*_(2,40)_ = 8.86, *p* = 0.001, η*^2^* = 0.31. Pair-wise comparisons revealed that the reaction times of the THS (619 ms) and the THD targets (626 ms) were significantly faster than that of the NR targets (639 ms, THS vs. NR: *t*_(20)_ = -4.56, *p* = 0.001; THD vs. NR: *t*_(20)_ = -2.57, *p* = 0.055). However, there was no significant difference between the THS and the THD targets (*t*_(20)_ = -1.42, *p* = 0.51).

The mean accuracy was 96.1, 94.7, and 97.1%, respectively, for the THS, THD, and NR targets. The repeated measures ANOVA of the accuracy data revealed a marginally significant main effect of Target condition, *F*_(2,40)_ = 2.84, *p* = 0.071, η*^2^* = 0.12. Pair-wise comparisons revealed that there was no difference among the THS, THD, and NR targets (*ps >* 0.10).

#### ERP Data

**FIGURE 6:**
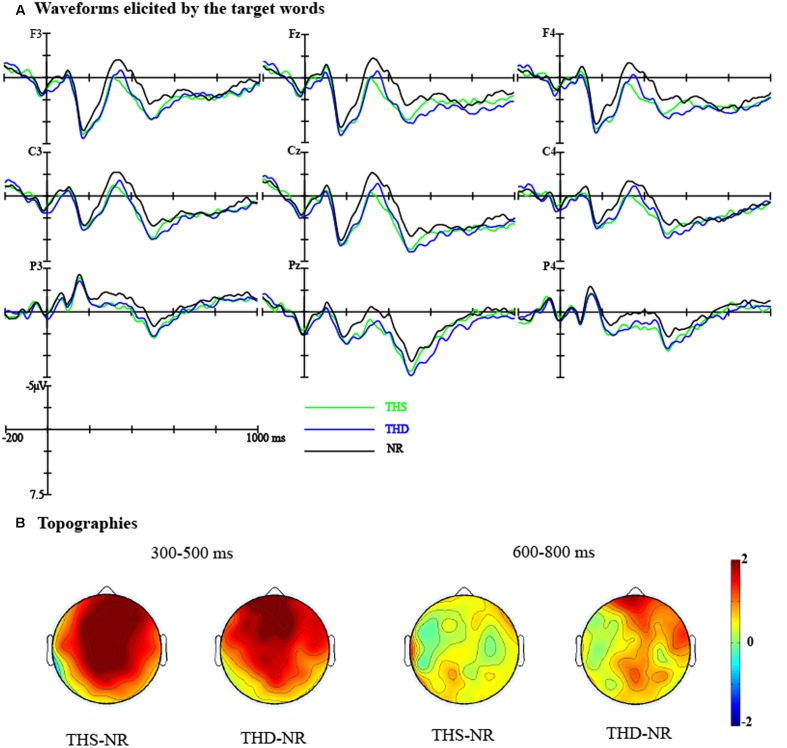
**(A)** Waveforms for THS, THD, and NR targets were presented at F3, Fz, F4, C3, Cz, C4, P3, Pz, and P4 electrodes; THS, thematically related word in the same episodes; THD, thematically related word in a different episode; NR, unrelated word. **(B)** Topographies showing the average amplitude voltage differences between the THS, THD targets, and NR targets, respectively.

The grand average waveforms elicited by the target words in different conditions at nine representative electrodes (F3, Fz, F4, C3, Cz, C4, P3, Pz, and P4) were presented in **Figure 6**. In view of the effects, two time windows were selected for statistic analyses: (1) the standard N400 time window: 300–500 ms; (2) A late positivity time window; 600–800 ms. Only significant effects containing the critical manipulation (Target condition) were reported.

##### The N400 time window

For the lateral electrodes, we found significant effects of Target condition [*F*_(2,40)_ = 27.92, *p* < 0.001, η*^2^* = 0.58] and an interaction between Target condition and Anteriority [*F*_(6,120)_ = 6.42, *p* < 0.001, η*^2^* = 0.24]. Separate ANOVAs for the Frontal, Central, Parietal, and Posterior regions revealed that the THS and THD targets elicited smaller N400s than the NR targets over the Frontal (THS vs. NR: *t*_(20)_ = 7.49, *p <* 0.001; THD vs. NR: *t*_(20)_ = 6.01, *p <* 0.001), Central (THS vs. NR: *t*_(20)_ = 7.24, *p <* 0.001; THD vs. NR: *t*_(20)_ = 5.25, *p <* 0.001), Parietal (THS vs. NR: *t*_(20)_ = 6.51, *p <* 0.001; THD vs. NR: *t*_(20)_ = 3.79, *p <* 0.01), and Posterior regions (THS vs. NR: *t*_(20)_ = 4.91, *p <* 0.001; THD vs. NR: *t*_(20)_ = 2.38, *p* = 0.083). In addition, there was no significant difference between the THS and THD targets over the Frontal, Central, Parietal, and Posterior regions (*ps >* 0.10).

For the midline electrodes, we also found significant effects of Target condition [*F*_(2,40)_ = 32.79, *p <* 0.001, η*^2^* = 0.62] and an interaction between Target condition and Anteriority [*F*_(6,120)_ = 3.59, *p <* 0.01, η*^2^* = 0.15]. Separate ANOVAs for the Frontal, Central, Parietal, and Posterior regions revealed that the THS and THD targets elicited smaller N400s than the NR targets over the Frontal (THS vs. NR: *t*_(20)_ = 5.28, *p <* 0.001; THD vs. NR: *t*_(20)_ = 7.44, *p <* 0.001), Central (THS vs. NR: *t*_(20)_ = 6.55, *p <* 0.001; THD vs. NR: *t*_(20)_ = 5.54, *p <* 0.001), Parietal (THS vs. NR: *t*_(20)_ = 7.17, *p <* 0.001; THD vs. NR: *t*_(20)_ = 4.51, *p* = 0.001), and Posterior regions (THS vs. NR: *t*_(20)_ = 4.85, *p <* 0.001; THD vs. NR: *t*_(20)_ = 3.10, *p <* 0.05). No significant difference between the THS and THD targets was found over the Frontal, Central, Parietal, and Posterior regions (*ps* > 0.05).

In sum, both the THS and the THD targets elicited smaller N400s than the NR targets over the whole scalp. In addition, there was no difference between the THS and the THD targets.

##### The LPC time window

For the lateral electrodes, there was a marginally significant interaction between Target condition and Hemisphere [*F*_(2,44)_ = 2.75, *p* = 0.075, η*^2^* = 0.11]. However, separate ANOVAs conducted to the left and right hemispheres revealed no significant effect of Target condition (*ps >* 0.10). No significant effect of Target condition was found for the midline electrodes either (*ps >* 0.10). In sum, there was no significant LPC difference between the THS, THD, and NR targets.

### Discussion

Experiment 2 revealed that the corresponding concepts of novel words primed both the THS and the THD targets, as indicated by both speeded RTs and smaller N400s. Moreover, the priming effect was comparable between the THS and the THD targets. The converging evidence from the behavioral and ERPs data suggests equally strong thematic relatedness for the THS and the THD targets with the corresponding concepts of the novel words. Therefore, the absence of the N400 priming effect as well as the LPC effect from the novel words to the THD targets in Experiment 1 can only be explained by the learning condition.

## General Discussion

In the current study, we examined whether the thematic knowledge of novel words could be derived from descriptive episodes and, if yes, whether the acquired thematic knowledge is dependent on the learning episodes. In Experiment 1, the ERP data showed that the learned novel words could prime both the corresponding concepts (CC targets) and the thematically related words in the same episodes (THS targets), as reflected by reduced N400s relative to the unrelated words (NR targets). However, the thematically related words in different episodes (THD targets) showed no priming N400 effect, instead they elicited a larger LPC compared to the unrelated words over frontal region. In addition, the CC and THS targets showed significant N400 effects compared to THD targets. In Experiment 2, the known words that represent the corresponding concepts of the novel words in Experiment 1 primed both the THS and the THD targets.

In the learning phase, the novel words for the first appearance elicited a larger N400 comparing to the second appearance. The N400 component has been proposed to reflect difficulty of semantic integration (Van Berkum et al., 1999; Chwilla et al., 2007; Kutas and Federmeier, 2011) or pre-activation of concepts (Van Petten et al., 1991; Besson et al., 1992; Mitchell et al., 1993). However, the N400 effect may partly reflect a repetition priming effect as the novel words were repeated during the second presentation (Misra and Holcomb, 2003; Grainger et al., 2012). In addition, the novel word in the first appearance elicited a significantly larger LPC comparing to the second appearance over the whole brain. Previous study have shown that the LPC reflects the retrieval of semantic and episodic information from long-term memory and the integration of the retrieved information into working memory (Van Petten et al., 1991; Batterink and Neville, 2011). In the learning phase, the participants were required to infer the novel words’ meaning on the basis of the descriptive discourse. Since the novel words were completely meaningless for the first appearance, the participants had to maintain the novel words on the working memory after they were presented for the first time. Meanwhile, they had to retrieve available prior information from long-term memory and link them to the current context in order to infer the meaning of the novel words. Thus, the LPC effect suggests that participants attempted to infer the meaning of the novel words and memorize the novel words after they were presented for the first time. Accordingly, the successful inference in the previous context facilitated the integration of the novel words into the context when they were presented for the second time, as indicated by the reduced N400.

For the behavioral data of Experiment 1 in the lexical decision task, no reliable priming effect was found between the novel words and the THS targets as well as the THD targets. However, the corresponding concepts of novel word showed clear priming effects for both the THS and the THD targets in Experiment 2. The lack of any priming effect on the RT data in Experiment 1 may be explained by the fact that the participants were not as familiar to the novel words as to the pre-existed words (i.e., the corresponding concepts of the novel words). Therefore, the participants spent more time to make responses in Experiment 1 comparing to Experiment 2. However, the priming effect was reflected by the response accuracy in Experiment 1. The participants recognized the corresponding concepts more easily than the thematically related targets following the novel words as the corresponding concepts might have been inferred and activated in the learning phase. However, upon seeing the thematically related targets, the participants may have to pay extra attention to process the relationships between the novel words and the thematically related words, which interfered the lexical judgements and induced more errors. In addition, the linking between the behavioral data with the ERP effect should be cautious (Luka and Van Petten, 2014), because the RT data, as an offline measurement, reflects a combination of several aspects of processing, such as perception, decision and motor operations. In the current study, the THS targets were thematically related to the learning episodes whereas the THD targets were not directly related to the learning episodes. As indicated by the ERP data, the THS targets elicited a smaller N400 effect whereas the THD targets elicited a larger LPC, indicating that participants were aware of the thematic associations between the novel words and the thematically related words from different scenarios in a late processing stage (Bouaffre and Faita-Ainseba, 2007). Therefore, they may pay more attention to process the relations between THD targets and learned novel words which resulted in a lower reaction time and higher error rate. In the lexical decision task, there could be both ERP effects and motor preparation within the critical time window. Behavioral results showed that the mean response time in the target conditions was about 650 ms and it did not differ among the four conditions. Therefore, any motor-related ERP components should be canceled out among the four target conditions. Moreover, any muscle artifact related to motor response has a higher frequency (above 30 Hz) comparing to the N400 and late positivity component (below 10 Hz). Therefore, it is very unlikely that the observed ERP effect was explained by the motor related activities.

As for the ERP data of Experiment 1, we found that the CC targets following the learned novel words elicited a smaller N400 as well as a larger LPC relative to the NR targets. The N400 effect was consistent with previous studies (Mestres-Missé et al., 2007; Borovsky et al., 2012; Chen et al., 2014). These results indicate that participants could successfully infer the meaning of the novel words from the descriptive episodes. The reduced N400 might reflect automatic spreading activation (Kiefer, 2002; Lau et al., 2008) and the LPC might reflect conscious awareness of semantic relationship between primes and targets (Bouaffre and Faita-Ainseba, 2007), suggesting that the corresponding concepts were automatically accessed after the participants read the newly learned words.

In addition, we found a reduced N400 for the THS targets relative to the NR targets over the whole brain. This suggests that the novel words could also prime the thematically related targets in the same learning episodes. During the learning phase, participants read the discourse passages and retrieved the episodes from long-term memory in order to infer the meaning of the novel words. During the inference process, although the THS targets were not directly presented, they might be activated due to their highly frequent co-occurrence with other information presented in the same episodes. Then during the testing phase, the recognition of the novel words facilitated the access to the THS targets and induced a reduced N400 (Lau et al., 2008). This result indicates that the thematic information of the novel words related to the learning episode could be learned via the inference of word meaning from descriptive episodes.

However, the thematic relations of novel words could not be generalized to other episodes, as we did not find any priming effect for the THD targets. Instead, we found that the THD targets elicited a larger late positivity relative to the NR targets over the frontal region. The late positivity have shown to relate to associations formed between semantically related words (Kim et al., 2012). It was proposed to reflect conscious awareness of semantic relations between primes and targets at a late processing stage (Bouaffre and Faita-Ainseba, 2007). Therefore, the late positive effect may indicate that the participants were only aware of the thematic associations between the novel words and the thematically related words from different scenarios in a late processing stage (Bouaffre and Faita-Ainseba, 2007). This effect is consistent with our previous study (Chen et al., 2014), in which we asked participants to learn novel words (e.g., *fangfen*) directly from mapping the novel words to given concepts (e.g., *turtle*). We found that the unlearned but feature-related targets (e.g., *long-lived*) also produced a late positive effect after such learning. Overall, the late positive effect might reflect delayed awareness of the semantic relations between the primes and the targets.

The absence of a priming effect of the THD targets and the presence of a priming effect between the THS and THD targets provide us some insight into the thematic organization of the novel words. The pre-test of the THS and THD targets suggests that they were equally related to the corresponding concepts of the novel words, which was further demonstrated by the comparable priming effects between the THS and THD targets with the corresponding concepts of the novel words in Experiment 2. However, the ERP data of Experiment 1 showed that the learned novel words could only facilitate the access of the thematically related words in the same episodes as the learning episodes. These results suggest that the thematic knowledge might be organized based on separate episodes. That is to say, although people tend to integrate thematically related words into an episode (Wisniewski and Bassok, 1999), the thematically related words of a concept that belong to different episodes might be represented as separate clusters. Therefore, the awareness of the thematic relations between the novel words and the concepts from different episodes might be mediated by the corresponding concepts of the novel words, leading to a late positive effect between the novel words and the thematically related words from different episodes. This conclusion is supported by other studies showing that only the novel word list that shared the same event or theme could be better recalled (Tinkham, 1997; Khayef and Khoshnevis, 2012; Mirjalili et al., 2012). Overall, the thematic knowledge of novel words is organized via separate scenarios or events, which is represented in a clustered manner in the semantic network.

Moreover, the CC targets elicited a larger N1 than the THS targets, which was distributed over the posterior region in midline and right hemisphere. In addition, the CC targets elicited a smaller P2 than the THS and NR targets over the posterior regions. Previous studies found that familiar words elicited a larger N1 than unfamiliar words (Proverbio et al., 2006, 2007). Also, the posterior P2 component was found to be susceptible to attention (Anllo-Vento and Hillyard, 1996). Take together, the larger N1 and smaller P2 elicited by the CC targets suggest that the corresponding concepts might have been pre-activated when the participants read the novel words, so that the CC targets attracted less attention than the THS and NR targets. These effects provide further support to our finding that the participants successfully inferred the meaning of novel words from the learning discourse passages.

In summary, the participants actively inferred the meaning of the novel words from descriptive discourse passages. The learned novel words could prime both the corresponding concepts and the thematically related words involved in the same episodes. Nevertheless, the newly learned words could not prime the thematically related words from different episodes. These results suggest that the thematic knowledge of novel words is represented in a clustered manner and that the acquisition of the thematic relations is largely dependent on learning contexts.

## Ethics Statement

The Institutional Review Board of the Institute of Psychology, Chinese Academy of Sciences. First, the participants were informed about the purpuse of the experiment: You have been asked to participate in a behavioral research study that focuses on neural mechanisms of Cognitive Control. We would like your permission to enroll you as a participant in this research study. Second, they knew about the procedure of the experiment. Declared as following: You will be asked to complete a series of tasks that measure your attention responses. Before the real task, you will be trained to make sure that you understand and can follow the task instructions. During this task, you will be asked to view visual stimuli such as words on the screen. Your task is to respond stimuli according to the task instructions by pressing a key corresponding to a certain stimulus or decision choice. The whole study will take about one and half hour. Third, they were informed about the risks and discomforts: There is no known risk and side effect beyond those that you usually encounter everyday. Then, they were informed that the results of this study may be published in an academic journal/book or used for teaching purposes. However, their name or other identifiers will not be used in any publication or teaching materials without your specific permission. And their participation is voluntary, refusal to take part in the study involves no penalty or loss of benefits. Finally, after they signed the Informed Consent Form for Experimental Participants, they took part the formal experiment. The participants in the current study are normal populations.

## Author Contributions

MZ, SC, LW, XY, and YY have all contributed to the conception, analysis, interpretation, drafting, critical revision, and final approval of the manuscript for publication.

## Conflict of Interest Statement

The authors declare that the research was conducted in the absence of any commercial or financial relationships that could be construed as a potential conflict of interest. The reviewer ARB and handling Editor declared their shared affiliation, and the handling Editor states that the process nevertheless met the standards of a fair and objective review.

## Footnotes

1 http://www.lsa.url.tw/modules/lsa/

2 We originally tested 23 participants while the comparison between THS and THD was not significant after correcting for multiple comparisons (*t*(22) = -2.28, *p* = 0.20, corrected for 6 pairs of comparisons). Then we tested six more participants in order to increase the statistical power on the basis of the power analysis of an independent dataset (Ding et al., 2017).
